# Mapping domain structures near a grain boundary in a lead zirconate titanate ferroelectric film using X-ray nanodiffraction

**DOI:** 10.1107/S1600576724009026

**Published:** 2024-10-29

**Authors:** Stanislav Udovenko, Yeongwoo Son, Pannawit Tipsawat, Reilly J. Knox, Stephan O. Hruszkewycz, Hanfei Yan, Xiaojing Huang, Ajith Pattammattel, Marc Zajac, Wonsuk Cha, Darren C. Pagan, Susan Trolier-McKinstry

**Affiliations:** a Department of Materials Science and Engineering and Materials Research Institute Pennsylvania State University State College Pennsylvania USA; b Materials Science Division Argonne National Laboratory Lemont Illinois USA; c National Synchrotron Light Source II Brookhaven National Laboratory Upton New York USA; d X-ray Science Division Argonne National Laboratory Lemont Illinois USA; Shiv Nadar Institution of Eminence, India

**Keywords:** nanodiffraction, ferroelectrics, thin films, *in situ*

## Abstract

The effect of an electric field on local domain structure near a 24° tilt grain boundary in a 200 nm-thick Pb(Zr_0.2_Ti_0.8_)O_3_ bi-crystal ferroelectric film was probed using synchrotron nanodiffraction. The bi-crystal film was grown epitaxially on SrRuO_3_-coated (001) SrTiO_3_ 24° tilt bi-crystal substrates. From the nanodiffraction data, real-space maps of the ferroelectric domain structure around the grain boundary prior to and during application of a 200 kV cm^−1^ electric field were reconstructed. In the vicinity of the tilt grain boundary, the distributions of densities of *c*-type tetragonal domains with the *c* axis aligned with the film normal were calculated on the basis of diffracted intensity ratios of *c*- and *a*-type domains and reference powder diffraction data. Diffracted intensity was averaged along the grain boundary, and it was shown that the density of *c*-type tetragonal domains dropped to ∼50% of that of the bulk of the film over a range ±150 nm from the grain boundary. This work complements previous results acquired by band excitation piezoresponse force microscopy, suggesting that reduced nonlinear piezoelectric response around grain boundaries may be related to the change in domain structure, as well as to the possibility of increased pinning of domain wall motion. The implications of the results and analysis in terms of understanding the role of grain boundaries in affecting the nonlinear piezoelectric and dielectric responses of ferroelectric materials are discussed.

## Abstract 2

Direct measurements have been taken of nanoscale domain structure in ferroelectric lead zirconate titanate around a grain boundary. Characterizing the evolution of this structure under an electric field is critical for predicting dielectric and piezoelectric response.

## 1. Introduction

Ferroelectric materials are extensively utilized in devices such as electromechanical actuators, transducers, sensors and multilayer capacitors. In many ferroelectrics, domain wall motion is an important contributor to the properties; this is typically referred to as the extrinsic contribution to the properties. In particular, domain wall motion entails the redistribution of polarization and strain, and so affects the dielectric and piezoelectric responses of the material (Tagantsev *et al.*, 2010 ▸; Jaffe *et al.*, 1972 ▸; Zhang *et al.*, 1994 ▸). For example, Fancher *et al.* (2017 ▸) reported that polarization reconfiguration due to 180° domain wall motion contributes >80% of the measured macroscopic polarization changes during switching in lead zirconate titanate (PZT) ceramics. Likewise, comparisons of X-ray diffraction and electrical polarization measurements in BaTiO_3_ ceramics showed that ∼70% of the large macroscopic dielectric permittivity in BaTiO_3_ (0.05 to 0.7 kV mm^−1^) arises from domain reversal (Fancher *et al.*, 2017 ▸). Jones *et al.* (2006 ▸) demonstrated that ∼34% of the measured piezoelectric *d*_33_ coefficient arose from motion of non-180° domain walls in PZT ceramics using *in situ* stroboscopic neutron diffraction data. Numerous other experimental and theoretical works have confirmed the important role that domain wall motion plays in macroscopic dielectric and piezoelectric response.

In bulk ferroelectrics, various crystalline defects such as dislocations, triple points and grain boundaries may act as either pinning centers or nucleation centers for domain walls (Gruverman *et al.*, 1997 ▸; Gao *et al.*, 2011 ▸; Gruverman, 2009 ▸; Jesse *et al.*, 2008 ▸). The role of grain boundaries is particularly complex. When grain boundaries act as pinning centers (Kalinin *et al.*, 2010 ▸) for domain walls, they lead to reduced electromechanical and dielectric responses (Damjanovic & Demartin, 1997 ▸; Randall *et al.*, 1998 ▸; Griggio & Trolier-McKinstry, 2010 ▸; Marton *et al.*, 2011 ▸; So *et al.*, 2005 ▸; Huey *et al.*, 2012 ▸). Conversely, new domains can nucleate at triple points and grain boundaries, inducing enhanced properties. However, there has been little direct quantitative characterization of the domain structure changes near a grain boundary and the mobility of domain walls. A more quantitative and statistically significant understanding of the way in which individual grain boundaries with varying properties (*e.g.* misorientation angles or the presence/absence of a coincident site lattice) influence the extrinsic contributions to the piezoelectric response is required for optimization of ferroelectric materials.

Given the numerous reports on collective domain wall motion in perovskite ferroelectrics, it is anticipated that the domain structure, not simply individual domain walls, will affect the pinning at grain boundaries. For example, the domain structures often are arranged to maintain strain and polarization compatibility across the grain boundary. This, in turn, means that 90° domain walls will not form near certain types of grain boundaries (Zhang & Bhattacharya, 2005 ▸). These distributed domain structures are predicted to respond collectively to applied fields. Indeed, phase-field modeling revealed a correlation between polarization switching in adjacent domains and coupling of the domain structure along grain boundaries (Choudhury *et al.*, 2005 ▸; Choudhury *et al.*, 2007 ▸). Experimentally, domain walls are widely known to have some level of continuity across grain boundaries according to microscopy techniques (Cao & Randall, 1996 ▸; Tsurekawa *et al.*, 2007 ▸; Mantri *et al.*, 2017 ▸) in both poled and unpoled ceramics, implying that the domain structures must move in some ways collectively.

Collective motion of domain walls was observed by switching spectroscopy piezoresponse force microscopy in polycrystalline Pb(Zr_0.52_Ti_0.48_)O_3_ films. Domain walls were found to undergo irreversible motion in clusters that ranged from ∼0.5 to 1 µm in size. This length scale considerably exceeded that of individual domain (10–30 nm) or grain sizes (∼50–150 nm). This behavior was attributed to correlated polarization switching (Bintachitt *et al.*, 2010 ▸). Band excitation piezoresponse force microscopy (BE-PFM) at lower fields can also be used to assess the irreversible-to-reversible Rayleigh ratio (Eitel, 2007 ▸) for the piezoelectric response under sub-switching conditions. Regions with high ratios correspond to areas where irreversible domain wall motion is favorable. It was shown that the cluster size for correlated motion of domain walls in PZT films was independent of whether the film was donor or acceptor doped (Peters *et al.*, 2023 ▸). BE-PFM results have also shown spatial clustering of nonlinearity in the piezoelectric coefficients of clamped polycrystalline and epitaxial ferroelectric films (Griggio *et al.*, 2012 ▸). Observation of clusters with increased nonlinear response with sizes significantly larger than the grain size suggests that the collective domain wall motion in different grains within a cluster contributes to Rayleigh behavior in PZT films (Trolier-McKinstry *et al.*, 2011 ▸).

Electron microscopy has been utilized for direct observation of domain wall motion in ferroelectric capacitors. For example, in {100}-oriented 100 nm-thick Pb(Zr_0.2_Ti_0.8_)O_2_ epitaxial films grown on (001) Nb-doped SrTiO_3_ substrates, *a* domains in the pristine sample were split into smaller *a* and *c* domains on poling, forming 90° strip domain structures. *c* domains are those with the polarization parallel to the substrate normal; domains with their *c* axis aligned within the sample plane are *a* domains. It was confirmed that 180° polarization switching contributed significantly to the out-of-plane polarization switching (Lee *et al.*, 2013 ▸). Ferroelectric domain patterns in Pb(Zr_0.8_Ti_0.2_)O_3_ and Pb(Zr_0.65_Ti_0.35_)O_3_ were analyzed using transmission electron microscopy (TEM) (Streiffer *et al.*, 1998 ▸). Electric fields at Pb(Zr_0.2_Ti_0.8_)O_3_ film/electrode interfaces have been observed by *in situ* TEM and are expected to affect the nucleation and growth rate of ferroelectric domains as well as the orientation and mobility of domain walls (Gao *et al.*, 2011 ▸). Though there are many other reports that use TEM to explore domain switching and nucleation and motion of domain walls (Li *et al.*, 2019 ▸), the statistical sampling of electron microscopy techniques tends to be small since only small volumes of material can be analyzed.

Piezoresponse force microscopy (PFM) has also been used to study extrinsic contributions to piezoelectric properties at individual grain boundaries and triple points; this approach can sample a larger number of domain walls interacting with a given grain boundary (Hennessey *et al.*, 2023 ▸; Marincel *et al.*, 2014 ▸; Marincel *et al.*, 2015*a* ▸; Marincel *et al.*, 2015*b* ▸). These studies established that the extrinsic contributions to the piezoelectric response vary in the range of tens of nm to nearly 1 µm away from microstructural features, depending on the grain boundary character. However, with PFM it is not possible to sample the domain structure through the volume of the film. As a result, changes in domain structure (and hence domain wall density) and in domain wall mobility (via pinning) could not be deconvolved in assessing the extrinsic contributions to piezoelectric and dielectric responses of ferroelectric material locally.

Additionally, synchrotron X-ray mapping techniques have been used to probe the domain structure of ferroelectric materials. Dark-field X-ray microscopy (DFXM) allows the distribution of strain in crystalline samples to be characterized (Yildirim *et al.*, 2020 ▸). Simons *et al.* (2018 ▸) demonstrated the feasibility of using DFXM to examine strain fields around domain walls in BaTiO_3_. However, current limitations on X-ray optics preclude distinguishing nanoscale domains in ferroelectrics. Furthermore, DFXM is generally limited to fixed fields of view of tens of µm, again dictated by optics. Beyond DFXM, angular splitting and the intensity of Bragg reflections have been utilized to determine the population of ferroelectric domains in the rhombohedral phase of Pb(Zr_0.976_Ti_0.024_)O_3_ single crystals. On the basis of the distribution of diffuse scattering, the positions of antiphase domain boundaries were identified (Udovenko *et al.*, 2018 ▸; Vakhrushev *et al.*, 2021 ▸). Nanodiffraction-based techniques such as scanning transmission X-ray microscopy and X-ray nanodiffraction are utilized for examination of fine structure features of nanocrystals and nanoscale devices (Chayanun *et al.*, 2019 ▸; Hruszkewycz *et al.*, 2011 ▸). *In situ* variation of sample environments (such as temperature, electric field) provides additional insight into the origin of material properties (Chayanun *et al.*, 2019 ▸; Sood *et al.*, 2021 ▸; Gamage *et al.*, 2024 ▸).

This paper explores the evolution of nanoscale domain structure in PZT piezoelectric bi-crystal thin films using synchrotron nanodiffraction. A custom sample assembly design is presented along with examination of PZT bi-crystal domain structure evolution under an applied electric field. The *in situ* nanodiffraction results are found to be heavily influenced by the domain structure of the as-deposited condition.

## 2. Sample preparation and characterization

**Figure 1:**
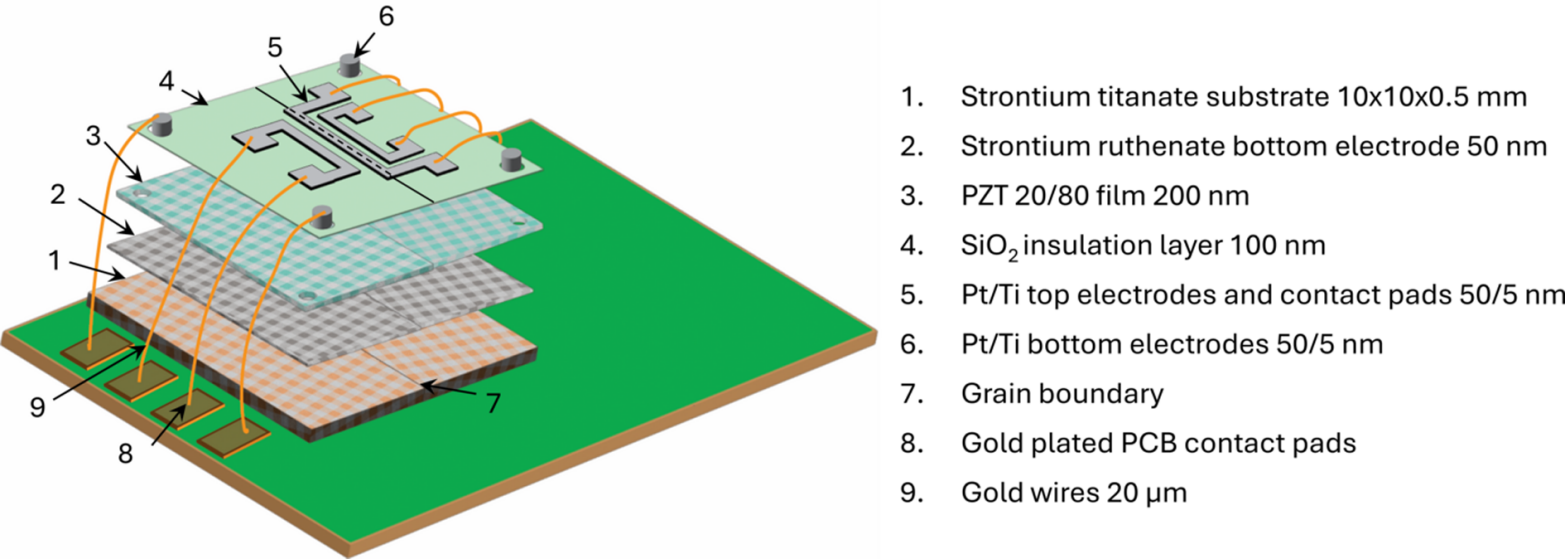
Labeled, exploded view of the sample assembly.

A custom sample assembly was designed for *in situ* application of an electric field to bi-crystal PZT specimens during nanodiffraction experiments. Fig. 1 ▸ shows a labeled, exploded view of the complete sample assembly.

The PZT bi-crystal studied in this work was grown on (001) SrTiO_3_ (STO) 10 × 10 × 0.5 mm bi-crystal substrates (MTI Corp.) with an in-plane tilt misorientation angle of 24°. Epitaxial films were deposited on the bi-crystal substrate via pulsed laser deposition with a KrF excimer 248 nm laser (Lambda Physik Complex Pro). First, a 50 nm SrRuO_3_ (SRO) bottom electrode was deposited from a ceramic target (Kojundo Chemical Lab. Co. Ltd) using a laser energy density of 1.5 J cm^−2^, a laser pulse frequency of 4 Hz, a substrate temperature of 665°C, a target–substrate distance of 6.7 mm and a chamber oxygen pressure of 120 mTorr. The deposition of the bottom electrode was followed by deposition of a Pb(Zr_0.2_Ti_0.8_)O_3_ (PZT20/80) film also from a ceramic target. To prepare the target, raw materials (Pb_3_O_4_, ZrO_2_, TiO_2_) in Pb:Zr:Ti mol% ratios of 60:10:40 were ball-milled and the resulting powder was calcined at 900°C for 4 h. The target was formed from the ball-milled powder using a cold isostatic press for 1 min at a pressure of 30 MPa, then sintered at 1050°C for 2 h. Deposition of a 200 nm PZT20/80 film was performed at a laser energy density of 1.5 J cm^−2^, a laser pulse frequency of 10 Hz, a substrate temperature of 600°C, a target-to-substrate distance of 6.2 mm and a chamber oxygen pressure of 85 mTorr. This process produced a sample in which the grain boundary propagated from the substrate through both epitaxial films.

To minimize the possibility of an electrical short to the bottom electrode for the large contact pads, an SiO_2_ layer was patterned over the top surface of the sample using the lift-off method. For the lift-off re-entry profile, LOR 5A photoresist was initially spun at 4500 RPM for 45 s and baked at 180°C for 3 min. This was followed by spinning SPR3012 photoresist at 4500 RPM for 45 s and baking it at 95°C for 1 min. The resist stack was exposed at 220 mJ cm^−2^ and developed in CD26 for 80 s. The sample was then ashed. Subsequently, a 100 nm thick SiO_2_ insulator layer was deposited by an electron beam evaporator (Kurt J. Lesker Lab-18) at a rate of 2 Å s^−1^ at room temperature. The sample was finally soaked in a PRS-3000 bath at 80°C to lift off the SiO_2_.

To expose the SrRuO_3_ bottom electrode for electrical contact, a 2.5 µm thick photoresist (SPR955) was spun on the sample at 2500 RPM and then baked for 1 min at 105°C. Subsequently, the photoresist was exposed with the desired pattern using a Heidelberg Instruments MLA-150 direct write tool at 400 mJ cm^−2^; the resist was developed in CD26 for 90 s. Before etching, the sample was cleaned with oxygen plasma for 2 min with 200 sccm of O_2_ and 50 sccm of He at 550 mTorr and 200 W radio frequency (RF) power. The PZT thin film was then patterned using an ULVAC NE-550 inductively coupled plasma–reactive ion etch tool. Etching was done using a chamber pressure of 3.8 mTorr with 10 sccm of Ar, 7 sccm of CF_4_ and 3.5 sccm of Cl_2_ at 600 W RF power, a bias power of 150 W, and an etching time of 150 s. Following this, the photoresist was stripped by immersing the sample in a PRS-3000 bath at 80°C.

Patterning of the strip-shaped top electrodes was done using the MLA-150 direct write tool after alignment to the grain boundary via an optical microscope. Finally, the top and bottom electrode layers were deposited using a DC magnetron sputter tool (Kurt J. Lesker CMS-18) and patterned using the lift-off method. The top electrode consisted of 5 nm of Ti and 50 nm of Pt; the depositions were carried out at room temperature without breaking vacuum. Active electrodes measuring 2700 × 5 µm were prepared along the center of the PZT grain boundary. Square contact pads (500 × 500 µm) and contact traces connecting the strip electrodes to the contact pads ran on top of the SiO_2_ layer. The samples were then affixed to custom printed circuit boards (PCBs) using silver paste.

**Figure 2:**
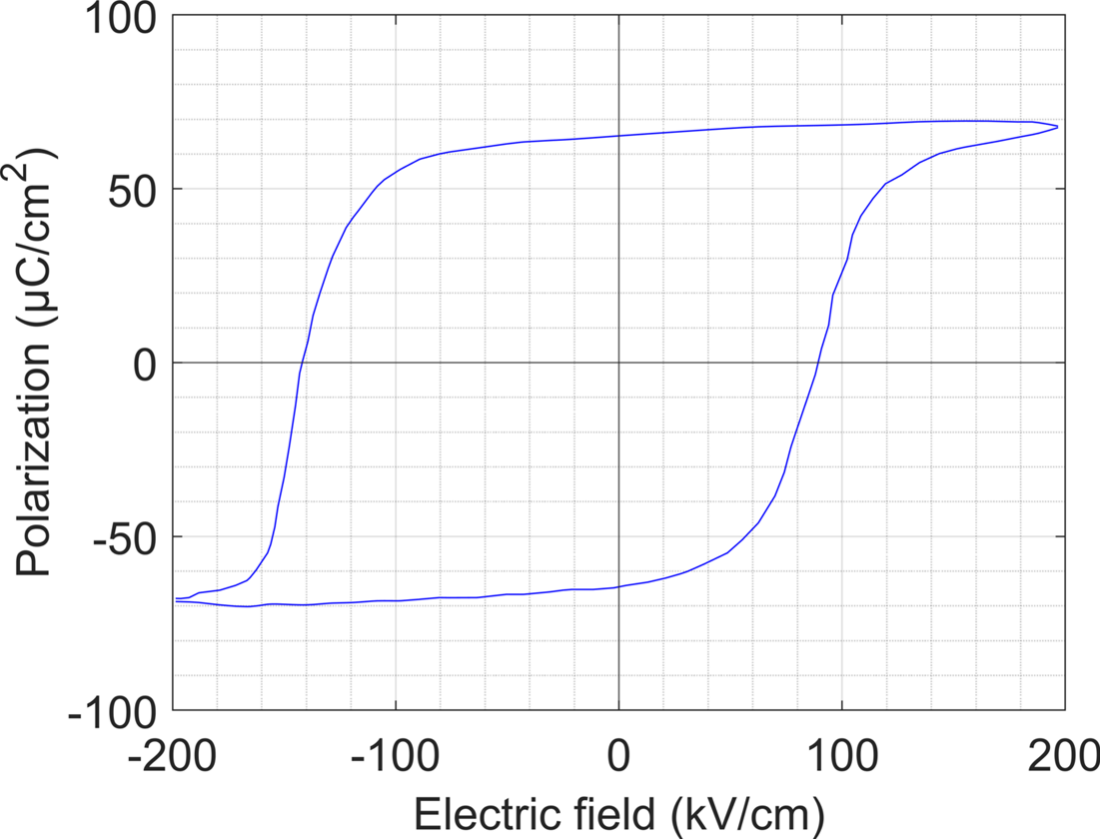
PZT20/80 film polarization – electric field hysteresis loop measured at 20°C and 10 kHz.

After the electrodes were deposited, electrical characterization of the sample was performed on an additional round electrode placed away from the grain boundary. Fig. 2 ▸ shows the polarization versus electric field hysteresis loop of the sample measured at 10 kHz at a temperature of 20°C. As the hysteresis loop shows, the positive and negative coercive fields 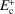 and 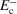 were 90 and −142 kV cm^−1^, respectively, and the remanent polarization was 65 µC cm^−2^. The negative bias indicates an internal field 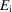 of −26 kV cm^−1^ calculated using equation (1) [following Akkopru-Akgun *et al.* (2019 ▸)],
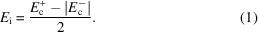
The measured sample capacitance indicated a dielectric constant of ∼136 and a loss tangent of 0.05. Internal electric fields, such as that observed in the tested film, are known to stabilize the domain structure against weak applied DC fields (Bassiri-Gharb *et al.*, 2007 ▸). However, in the synchrotron experiment, large DC fields were applied to the sample, so this domain structure stabilization effect should be negligible.

**Figure 3:**
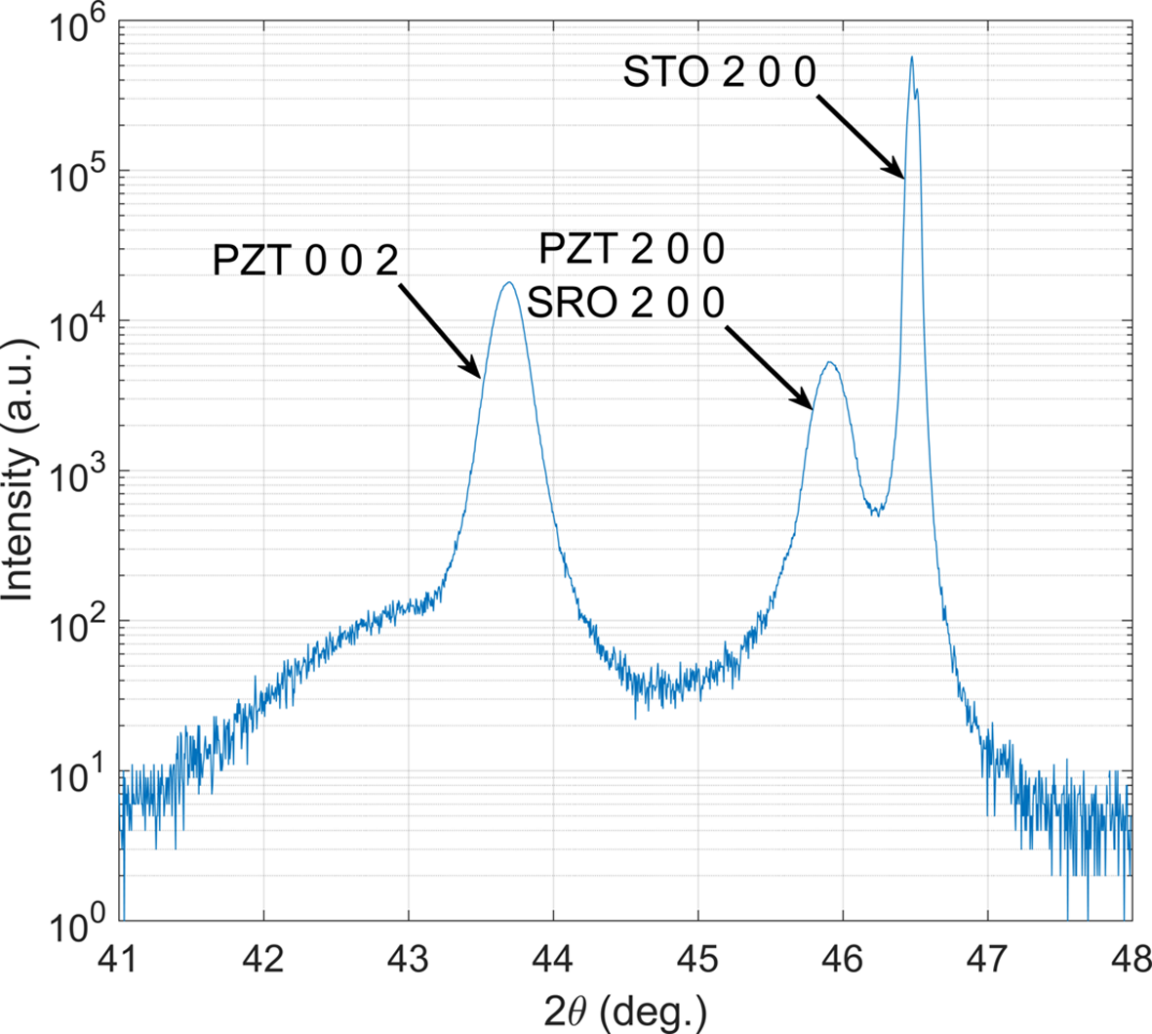
1D diffraction pattern of intensity versus 2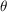 from the PZT/SRO/STO film stack on a logarithmic scale.

Laboratory-source X-ray diffraction (X’Pert^3^ MRD diffractometer with Cu *K*α radiation) performed in a reflection geometry confirmed that the PZT films were phase-pure perovskite and had tetragonal symmetry (*P*4*mm* space group), as expected, on the basis of the Zr/Ti ratio. Fig. 3 ▸ shows the intensity versus 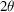 angle in a region containing STO, SRO and PZT20/80 peaks. Note that the PZT 200 and SRO 200 peaks overlap. The films are structurally relaxed as seen from the relatively broad diffraction peaks, which is expected due to the significant lattice mismatch between the three materials at room temperature. In Fig. 3 ▸, the 002 PZT peak has significantly higher intensity than the overlapped 200 peaks, indicating that the film is predominantly composed of domains with their *c* axis aligned with the sample surface normal. This higher volume fraction of *c* domains is consistent with the PZT film being under finite compressive stress since the thermal expansion coefficient of SrTiO_3_ is higher than that of PZT20/80. As described elsewhere (Tuttle *et al.*, 1992 ▸; Funakubo *et al.*, 2012 ▸; Coleman *et al.*, 2019 ▸), films of this composition cooled through the ferroelectric transition temperature under compressive stress tend to be predominantly composed of out-of-plane-oriented *c* domains.

**Figure 4:**
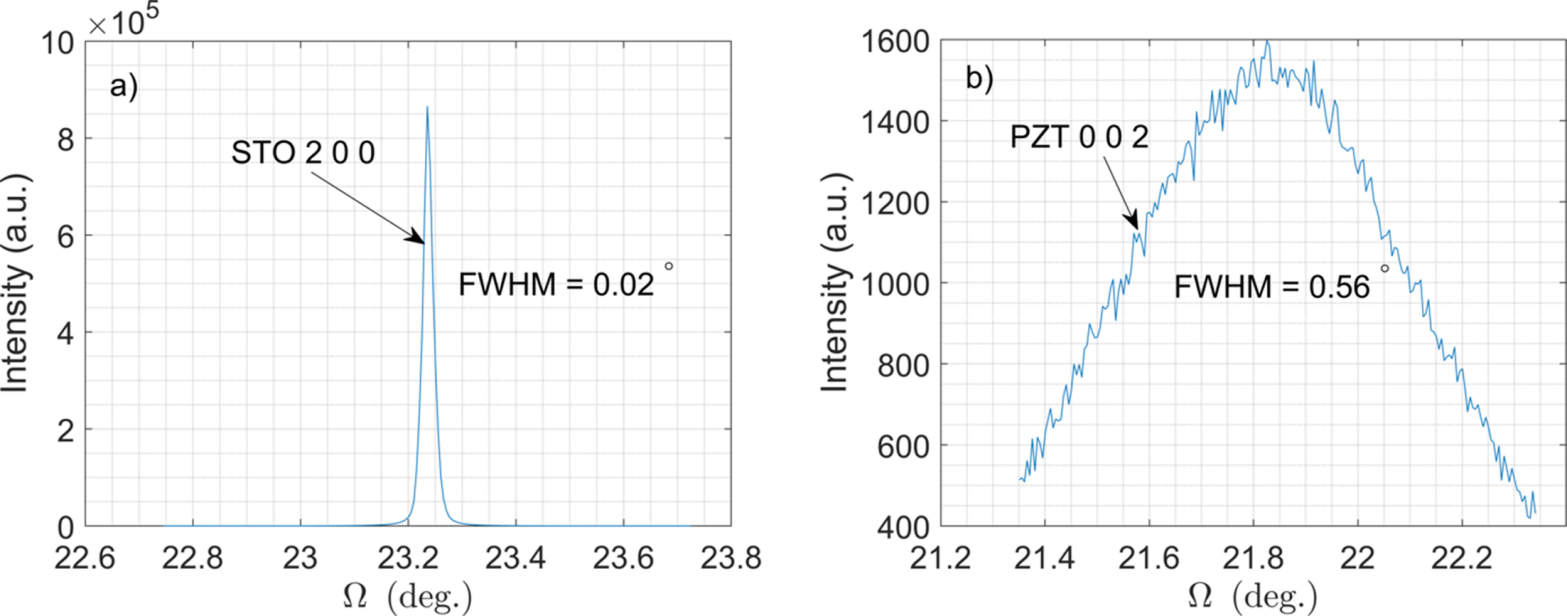
Rocking curve of 200 diffraction peak of STO substrate (*a*) and rocking curve of 002 diffraction peak of PZT film (*b*).

Crystallinity (or mosaicity) of the film and substrate was determined by rocking the sample across the angle 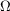 to measure the distribution of intensity perpendicular to the radial direction of reciprocal space. Fig. 4 ▸(*a*) presents a rocking curve related to the 200 STO substrate peak, while Fig. 4 ▸(*b*) shows a rocking curve for the 002 PZT peak. In both figures, the full width at half-maximum (FWHM) values of the peaks are labeled. The FWHM of the PZT 002 peak confirms the reasonable crystallinity of the deposited PZT film.

## 3. Synchrotron nanodiffraction measurments

**Figure 5:**
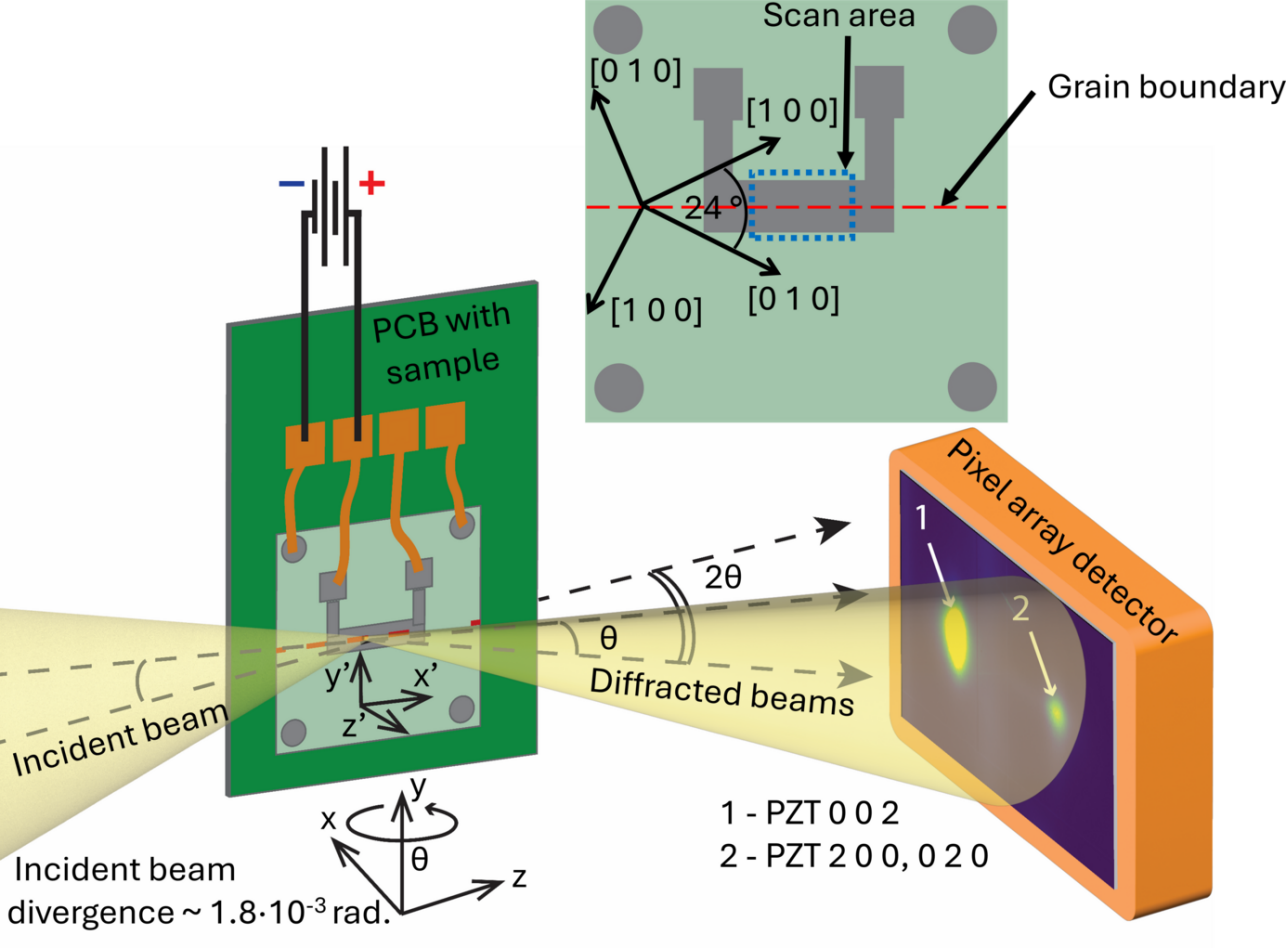
Experimental setup for the nanodiffraction measurements. The laboratory coordinate system is labeled *xyz*; the sample coordinate system is labeled *x*′*y*′*z*′. Diffracted intensity is emitted at an angle 2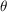 from the incoming X-ray beam. The 002 PZT (1) and 200 PZT (2) diffraction peaks on the pixel array detector are labeled.

The synchrotron experiment was performed at the 3-ID Hard X-ray Nanoprobe beamline at Brookhaven National Labora­tory Synchrotron Light Source II. A schematic layout of the experimental and sample geometry is provided in Fig. 5 ▸ with the laboratory (*xyz*) and sample (*x*′*y*′*z*′) coordinate systems labeled. The incoming X-ray beam travels along the *z* axis. The angle 2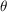 (twice the Bragg angle) is the angle between the incoming and diffracted beams. Measurements were performed in a horizontal scattering geometry with the PZT film normal placed in the horizontal scattering plane. The relationship between the scattering angle and lattice plane spacing is given by Bragg’s law,
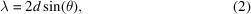
where λ is the wavelength of the incoming X-ray beam and *d* is the lattice plane spacing. The sample was placed in an He-filled chamber along with the focusing X-ray optics. Focusing was achieved by a Fresnel zone plate with a 30 nm outermost zone width, which focused the incoming beam to about 37 × 37 nm with a numerical aperture of 1.8 × 10^−3^ rad. The incoming X-ray beam energy was 11.6 keV (λ = 1.069 Å). The working distance between the specimen and the order sorting aperture was approximately 10 mm. The specimen sat on a positioning stack consisting of a hexapod for coarse specimen alignment and sample rotation that supported a set of piezoelectric motors for fine specimen translations and sample scanning. The sample was rotated by angle 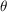 about the *y* axis.

Three different detectors were utilized during the experiment: a 2D pixel array detector for diffraction measurements (Merlin4X, 512 × 512 pixels, 55 µm pixel size), an energy-dispersive silicon drift detector for fluorescence (Vortex ME3), and an imaging detector comprising a coupled scintillator and optical camera (Prosilica) to roughly position the sample assembly with respect to the beam. The imaging detector was used to locate a corner of the sample substrate. From there, the sample was translated such that the beam was approximately in the center of the bi-crystal PZT film. The top electrode was then located by performing a 2D grid scan over the specimen surface while collecting fluorescence data. The electrode position was precisely determined by the Pt fluorescence signal. It is noted that the large sample size relative to the typical sample size for this X-ray microscope precluded the ability to place the diffraction volume directly over the rotation axis, so the sample unavoidably precessed and shifted during rotation about the *y* axis.

Once the beam was aligned to the electrode (and the underlying grain boundary), the diffraction detector was positioned to subtend the PZT 200 and 002 diffraction peaks. First, the sample was rotated to 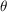 = 15° to place the STO 002 lattice planes into the diffraction condition. The detector was shifted to a sample-to-detector distance of 500 mm (sufficient to separate the 200 and 002 PZT diffraction peaks) and then moved horizontally until the intensity from the STO 200 peak was measured. The detector was next shifted until the SrTiO_3_ 200, PZT 200 and PZT 002 peaks could all be simultaneously captured on the detector panel.

**Figure 6:**
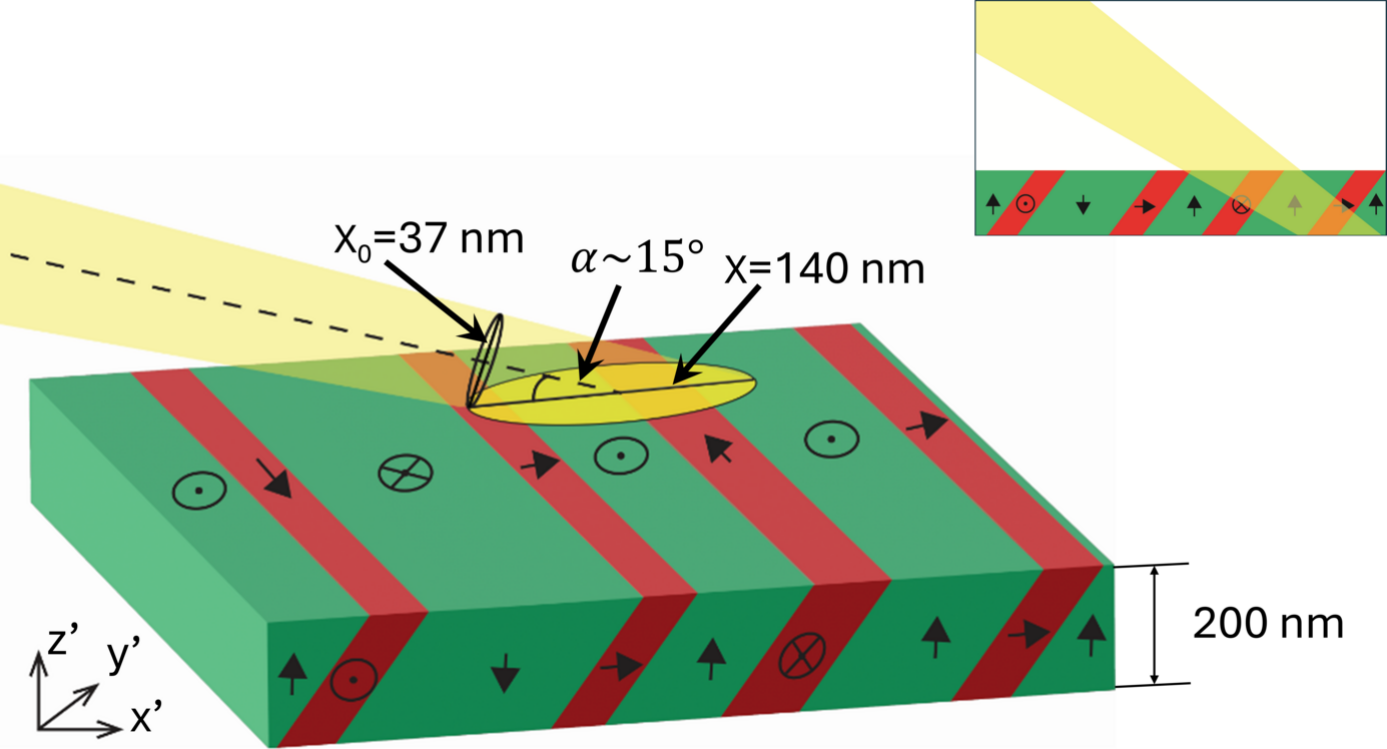
Schematic illustration of the incident beam footprint transformation according to equation (3) and its effect on the illuminated diffraction volume. Green corresponds to *c* domains and red to *a* domains. Inset shows the path of the X-ray beam through the film.

Of note is the footprint of the incoming X-ray beam on the specimen, as this plays a major role in defining the diffraction volume size, spatial resolution and illumination of domains, along with the interpretation of diffracted intensity magnitudes. Given the angle of incidence, the beam footprint had an oval shape with a major axis determined by
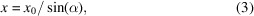
where *x* is the footprint, 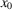 describes the normal incidence beam size and α is the angle of incidence (in this case equivalent to the angle 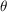). For these measurements, α ≃ 15°, which extends the beam by a factor of 4 along *x*′, producing a beam footprint of 143 × 37 nm along *x*′ and *y*′. Fig. 6 ▸ illustrates the transformation of the beam footprint according to equation (3) and the resulting diffraction volume. Due to the film thickness, domain wall inclination, angle of incidence and finite beam size, multiple domain types can be illuminated despite the nanofocused X-ray probe.

**Figure 7:**
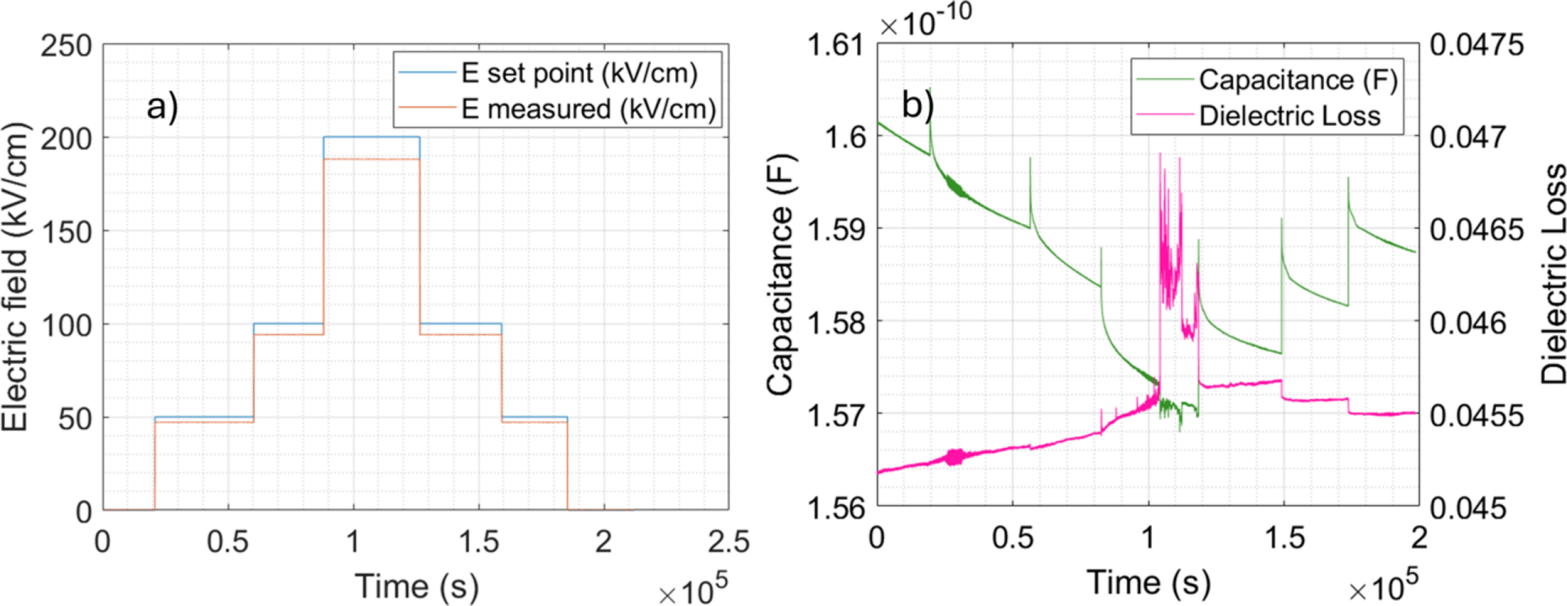
Electric field set point (blue line) and measured electric field in sample (orange line) (*a*); capacitance (green line) and dielectric losses of sample (purple line) (*b*). The AC excitation signal used for the permittivity measurements was 50 mV with a frequency 2 MHz.

The system for *in situ* application of the electric field included a Keysight Keithley 4980A precision LCR meter, a Raspberry Pi 4B single-board computer, a 16-channel relay module (Sainsmart) and a power supply (Alitove). The system was controlled via the Raspberry Pi 4B and a custom Python script. The LCR meter served as a voltage source and enabled continuous measurement of voltage, capacitance and dielectric loss during application of the electric field. Electric fields were applied between the top and bottom electrodes along the *z*′ axis corresponding to the film normal direction (see Fig. 5 ▸). The LCR meter was connected to the sample through a relay module placed inside the sample chamber. The relay module controlled which electrode on the sample was active. Fig. 7 ▸(*a*) shows the set point and measured values of the electric field *E* as a function of time through the experiment. Upon increasing the electric field, the difference between the set value of the electric field and measured electric field increased due to an increase in the sample leakage current. Similarly, Fig. 7 ▸(*b*) shows the sample’s capacitance and dielectric losses. The capacitance peaked directly after switching of the electric fields. Dielectric losses grew with increasing electric field, while the region of high amplitude and frequency of oscillations of the dielectric loss observed at maximum electric field (200 kV cm^−1^) may indicate that the sample was close to electrical breakdown.

Diffraction scans were performed on the specimen at each increment of electric field (0, 50, 100, 200, 100, 50, 0 kV cm^−1^). However, the analysis here focuses on application of the peak field where the evolution of the domain structure was expected to be largest. A diffraction scan consisted of rotating the specimen across a sequence of 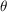 angles between the range of 14.5° and 16° in 0.25° increments. At each θ angle, diffraction patterns were measured on a grid of points in the *x*′–*y*′ plane. The grid spanned 6 × 5.1 µm with measurement spacing of 30 nm for a total of 200 × 170 (34000) diffraction patterns. Critically, the experimental configuration and sample design precluded registration of the specimen along the electrode/grain boundary as extra fiducials were not deposited. For this reason, shifts of the sample along *x*′ during application of the electric field or due to sample rocking could not be corrected, leading to motion of the scan region of the sample.

To reduce the size of the stored diffraction data, only a 509 × 289 pixel sub-region that contained the SrTiO_3_ 200, PZT 200 and PZT 002 peaks was saved. The peak intensity of the 200 PZT peak (corresponding to *a* domains) was measured at 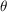 = 15.85° and the peak intensity of the 002 PZT peak (corresponding to *c* domains) was measured at 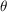 = 14.80°. Processing of the diffraction data was performed using a set of Python scripts described in more detail in the next section. Prior to processing, diffraction patterns were normalized by the incoming beam flux.

## 4. Analysis and results

### 4.1. Spatial distributions of scattering features

**Figure 8:**
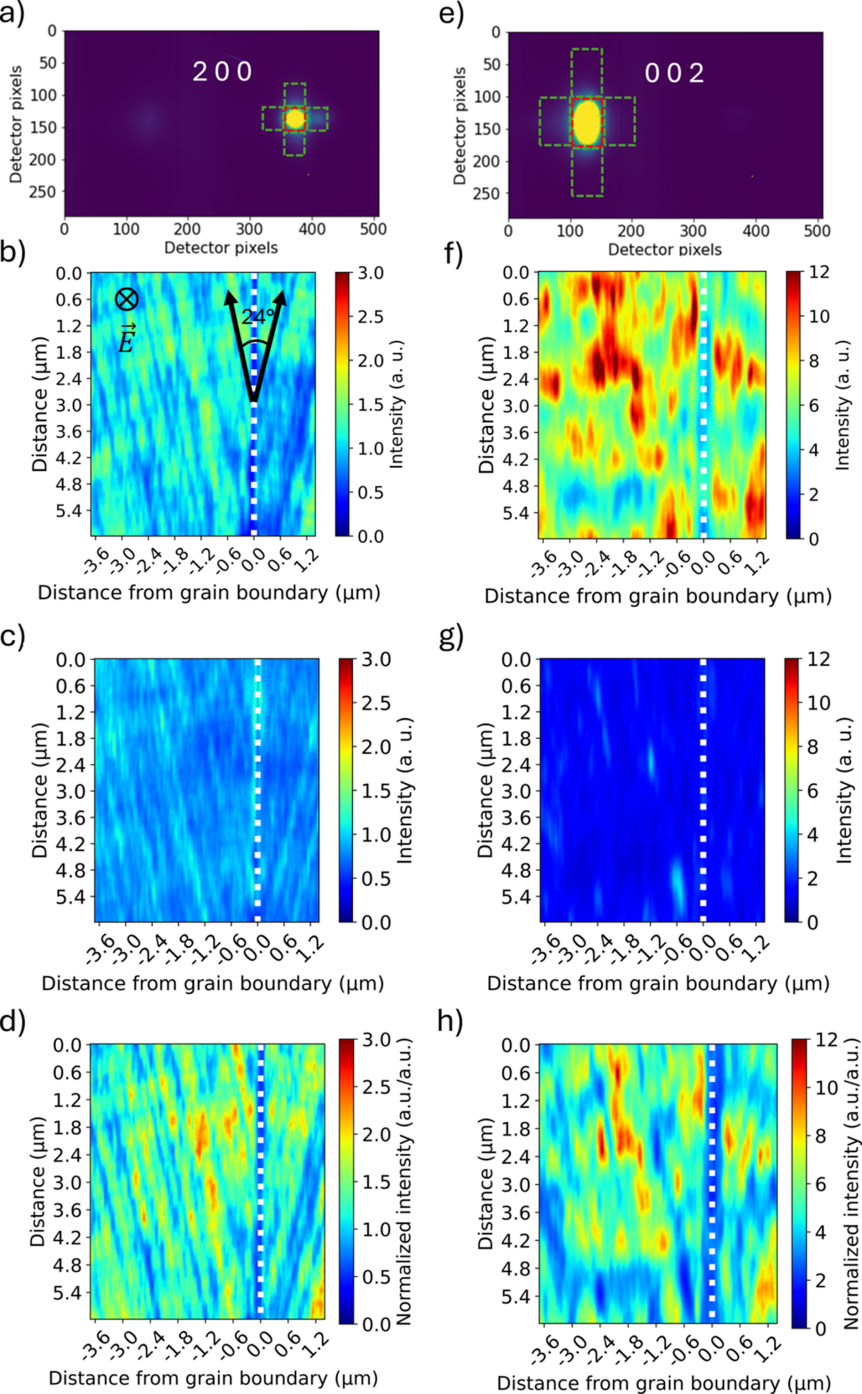
Representative diffraction patterns measured at 0 kV cm^−1^, including *a*-domain peaks (*a*) and *c*-domain peaks (*e*) with masks; distribution of primary peak intensity for *a* and *c* domains [(*b*), (*f*)]; distribution of diffuse scattering from microstructure features around *a*-domain peaks and *c*-domain peaks [(*c*), (*g*)]; distribution of primary intensities divided by diffuse scattering [(*d*), (*h*)]. Grain boundary position shown with white dotted lines. Indicated in (*b*) is the 24° angle between [100] directions on the STO bi-crystal on which the epitaxial PZT was grown.

To gain insight into the real-space domain structure, the series of diffraction patterns were processed to build spatial maps of various reciprocal-space intensity features. The diffraction images used to generate these maps at 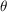 = 15.85° and at 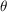 = 14.80° corresponded to the peak intensity of the 200 and 002 PZT diffraction peaks, respectively, which in turn were associated with scattering from *a* domains and *c* domains. The primary scattering at the center of the diffraction peak associated with the bulk of the illuminated domains was separated from diffuse scattering associated with distorted regions of the domains (*i.e.* regions near domain walls). Extraction of the intensity of interest from the diffraction patterns was performed using binary masks. Primary scattering (marked with red dashed boxes) and diffuse scattering (marked with green dashed boxes) mask regions are shown on representative images for scattered intensity from *a* domains and *c* domains in Figs. 8 ▸(*a*) and 8 ▸(*e*), respectively. In these diffraction images, the horizontal direction corresponds to the radial direction in reciprocal space associated with variation in lattice plane spacing, while the vertical direction corresponds to variation in lattice plane normal orientation.

The diffracted intensity within the masks was summed on each diffraction image and then mapped to the grid of measurement points in real space (200 × 170 points with 30 nm spacing). Figs. 8 ▸(*b*) and 8 ▸(*f*) show the summed intensity from the primary scattering masks around the PZT 200 and 002 peaks at 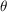 = 15.85° and 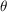 = 14.80°, respectively. In these figures [and Figs. 8 ▸(*c*), 8 ▸(*d*), 8 ▸(*g*), 8 ▸(*h*)], the maximum intensity of the color scale varies in order to improve feature contrast. Also note that these two real-space maps correspond to different regions along the electrode (due to specimen shifts during rocking). In Fig. 8 ▸(*b*), one can see a stripe structure that forms at angles close to 24°, which corresponds to the angle of in-plane misorientation of the bi-crystal sample film. Within each spatial map (140 × 37 × 200 nm), there is nonzero intensity at all points, indicating both *a* and *c* domains are generally present. However, for most points on the spatial maps, *c* domains are dominant. In Fig. 8 ▸(*f*), one can note the existence of ‘islands’ of regions of elevated intensity that correspond to the significantly increased presence of *c* domains and reduced presence of *a* domains.

Figs. 8 ▸(*c*) and 8 ▸(*g*) show the summed intensity from the same two scans in Figs. 8 ▸(*b*) and 8 ▸(*f*), but instead using the diffuse masks (marked with green dashed boxes). Taking into account the orientations of domain walls in bi-crystal PZT films with the same composition measured by PFM (Marincel *et al.*, 2014 ▸; Marincel *et al.*, 2015*a* ▸; Marincel *et al.*, 2015*b* ▸), one can suggest that the diffuse scattering is associated with the distorted crystal near domain walls. Increased diffuse scattering can also be observed near the grain boundary in Fig. 8 ▸(*c*), which is consistent with the primary contributor to the diffuse scattering being distorted regions of the crystal. Figs. 8 ▸(*d*) and 8 ▸(*h*) show the distribution of primary peak intensity divided by the distribution of intensity scattered by domain walls, providing a merged view of the domain composition and wall structure. By dividing primary scattering, mainly from the bulk of domains, by the diffuse scattering, mainly from the domain walls, further feature enhancement is achieved, allowing us to observe the domain structure present.

### 4.2. Domain structure characterization under an applied field

**Figure 9:**
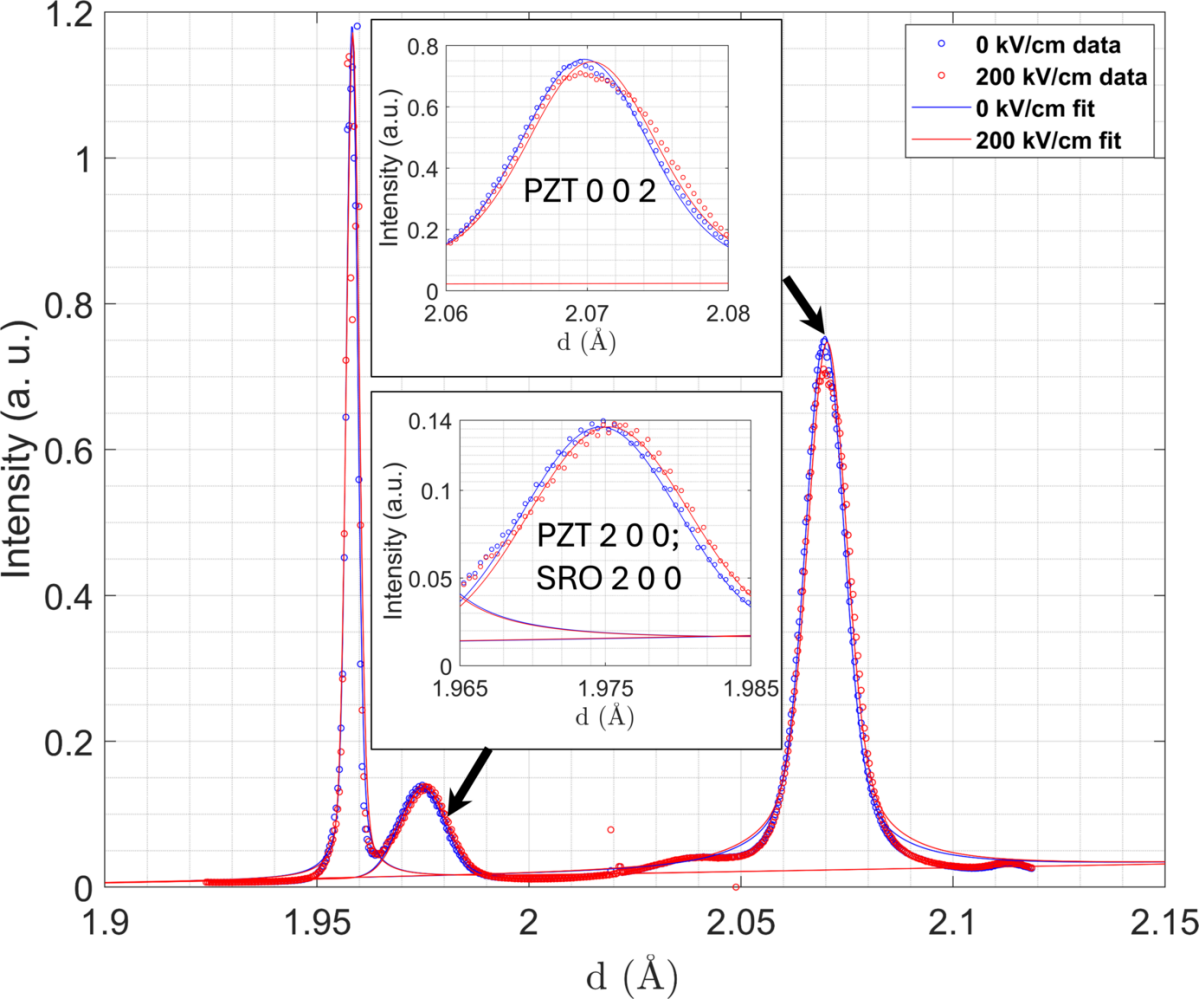
Integrated 1D diffraction patterns (circles) and corresponding peak fits (solid lines) from the PZT bi-crystal film collected at 0 and 200 kV cm^−1^ (blue and red colors, respectively).

The average response of all scanned regions as the sample was rocked in 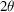 was evaluated to probe bulk domain switching and facilitate segmentation between regions primarily composed of *a* or *c* domains. 1D intensity line profiles were constructed by summing the 2D diffraction images perpendicular to the radial direction in reciprocal space to collapse the data onto the 2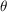 axis, and then summing across all scan points and 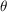 angles. Fig. 9 ▸ shows the summed intensity of the STO, SRO and PZT Bragg peaks versus lattice plane spacing [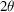 transformed to *d* using equation (2)] for both 0 and 200 kV cm^−1^ applied to the PZT bi-crystal film. As can be seen, there is a broadening of the PZT 200 and 002 diffraction peaks with applied field. This is believed to be from an increase in strain heterogeneity arising from the need to maintain deformation compatibility (Denis-Rotella *et al.*, 2020 ▸).

The 002 and 200 diffraction peaks were fitted with pseudo-Voigt functions to establish the mean position associated with the average lattice plane spacing 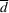 and the total integrated intensity *I*. Analysis of the PZT 002 (*c* domain) peak position showed the peak shift to higher lattice plane spacing values at an electric field of 200 kV cm^−1^ compared with zero field. The intrinsic piezoelectric strain 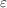 was calculated from the peak positions using the relationship
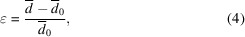
where 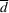 is the mean lattice plane spacing under an applied field and 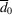 is the mean lattice plane spacing prior to application of a field. The intrinsic piezoelectric strain was calculated to be 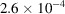, with the uncertainty of the strain determined from fitted peak positions estimated to be 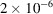 following Daymond *et al.* (2002 ▸). Using this strain, the value of *d*_33,f_ (where the subscript f denotes that this is a piezoelectric coefficient measured from a film) was evaluated as
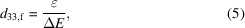
where 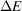 is the increase in applied electric field . This analysis gives 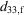 as 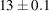
 pm V^−1^, which is commensurate with the value of 10 pm V^−1^ found in the literature (Xu *et al.*, 1999 ▸). A concurrent shift in the peak corresponding to *a* domains was attributed to the ‘passive’ elongation of *a* domains caused by the need to maintain deformation compatibility with adjacent *c* domains. This model is consistent with prior reports by Pramanick *et al.* (2011 ▸).

**Figure 10:**
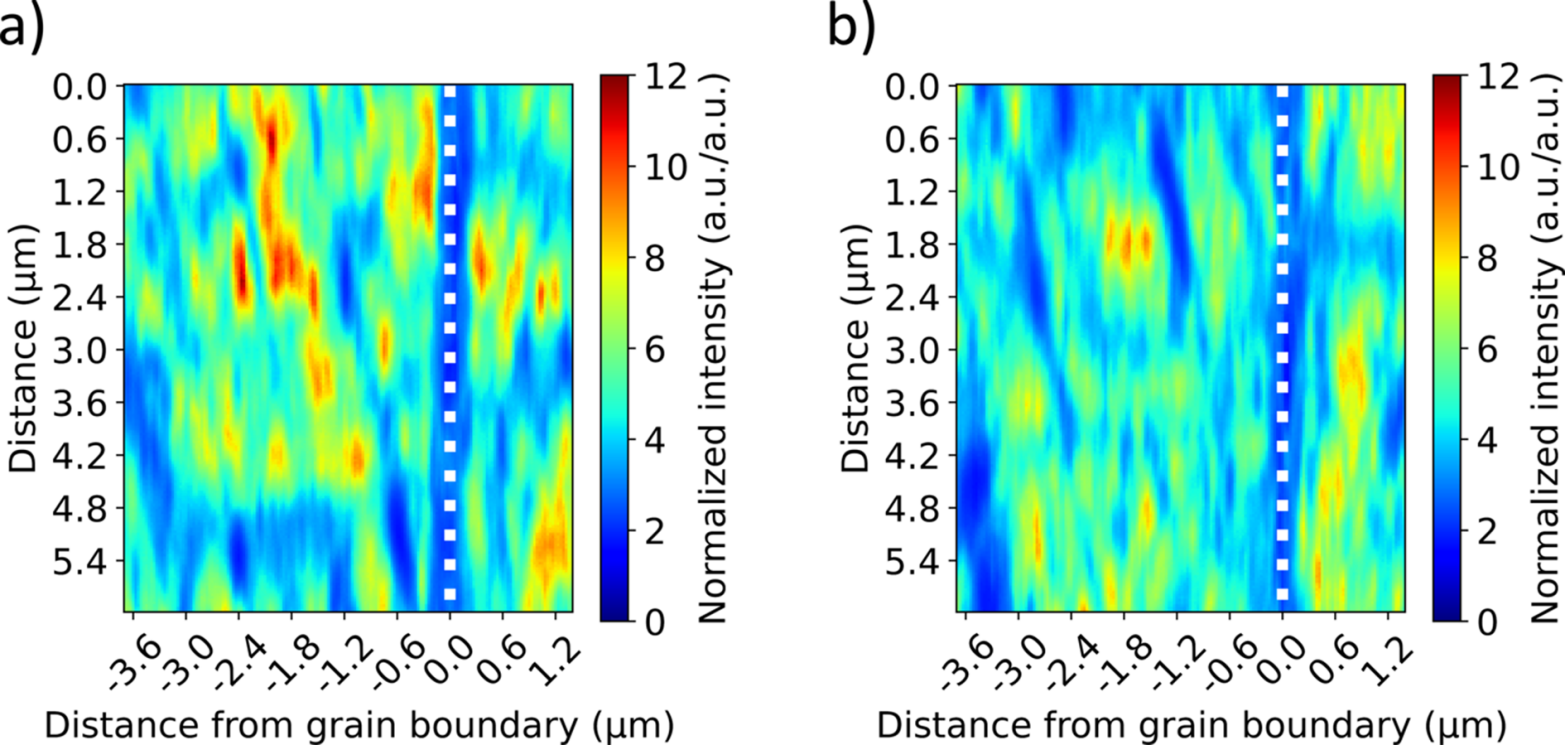
Primary scattering intensity maps associated with *c* domains at 0 and 200 kV cm^−1^ shown in (*a*) and (*b*), respectively. The white dotted line represents the position of the grain boundary.

The volume fraction of *c* domains, 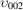, was calculated from the 1D line profiles in Fig. 9 ▸ using the formula [details given by Jones *et al.* (2005 ▸), Key *et al.* (2005 ▸)]
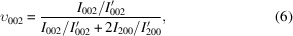
where 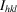 is the integrated intensity of the diffraction peak from the sample and 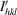 is the reference value of intensity from the powder diffraction data (Joseph *et al.*, 2000 ▸). The population of *c* domains with polarizations perpendicular to the film surface was close to 87% at both 0 and 200 kV cm^−1^. The relatively minimal change of the bulk volume fractions of the domains due to substrate constraints is noted; however, this does not preclude reconfiguration of the domain structure (as will be seen). Fig. 10 ▸ shows real-space maps corresponding to 0 kV cm^−1^ (*a*) and 200 kV cm^−1^ (*b*).

**Figure 11:**
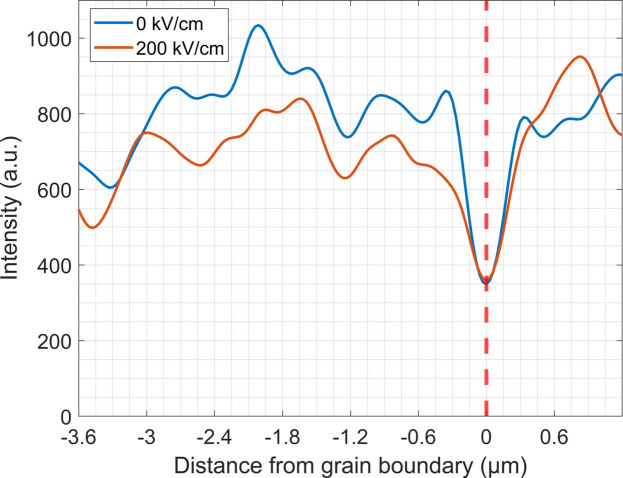
Diffracted intensity related to scattering of *c* domains [Figs. 10 ▸(*a*) and 10 ▸(*b*)] integrated along grain boundary direction (*x*′).

To analyze more closely *c* domains as a function of distance from the grain boundary and electric field, the intensity distributions shown in Fig. 10 ▸ were summed parallel to the grain boundary (vertical dotted line). Fig. 11 ▸ shows the diffracted intensity associated with *c*-domain density distribution perpendicular to the grain boundary at 0 kV cm^−1^ (blue line) and at 200 kV cm^−1^ (orange line). Clearly, away from the grain boundary, the density of *c* domains is relatively high, regardless of applied field. Also of note is a decrease in the intensity (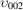) within ±150 nm of the grain boundary as determined from a 50% decrease in intensity associated with *c* domains. The high density of *c* domains in the sample volume (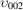) is consistent with the film being in a compressive stress state as described above. As was observed with the global volume fraction measurements, there is minimal change in the total intensity with an applied field. The small reduction of intensity with field, the reverse of the expected behavior, is due to the previously mentioned shift of the scanned region on the sample, not a decrease in *c*-domain volume fraction. The observed change in the domain state must be one of the contributors to reduced irreversible-to-reversible Rayleigh ratios at comparable bi-crystal boundaries reported by Marincel *et al.* (2014 ▸, 2015*a* ▸, 2015*b* ▸). This suggests that, in order to fully understand the role that domain walls play in the extrinsic contributions to the piezoelectric and dielectric responses, it will be important to understand not just the local Rayleigh behavior but also the local domain structure. Local diffraction methods provide a means to assess the latter quantitatively, in a way that is currently challenging to do with other techniques.

## 5. Conclusions

The domain structure in a 200 nm bi-crystal PZT20/80 film was probed via X-ray nanodiffraction around a 24° tilt grain boundary under a controlled out-of-plane applied electric field. It was found that the separation of primary and diffuse scattering around diffraction peaks facilitated identification of domain structure features. An analysis of the domain densities as a function of distance from the grain boundary revealed a significant drop in the volume fraction of *c*-type tetragonal domains to ∼50% within ±150 nm from the grain boundary. The high total volume fraction of *c* domains (∼87%) is due to compressive stress from the STO substrate. In addition, measurements showed that the *c* domains in the as-deposited sample evolved little during application of 200 kV cm^−1^ due to clamping by the substrate.

The measurements shown here highlight fundamental challenges for direct characterization of domain structure using nanodiffraction that require consideration. The combination of achievable beam footprint, through-thickness penetration and domain wall orientation (here parallel to {101} lattice planes) likely precludes isolation of single domains when probing realistic ferroelectric films (illustrated in Fig. 6 ▸). In the data presented, varying intensity associated with *c* domains was measured in every probed diffraction volume (see Fig. 10 ▸), indicating that *a* and *c* domains were generally always illuminated. Thus, for more quantitative measures of domain composition in each diffraction volume, simultaneous mapping of real space and reciprocal space through measurements of rocking curves at each measurement point is necessary. With these measurements, local volume fractions, in addition to quantities such as local strain state, would be probed. These measurements are possible but require position stability or re-registration that was not performed here due to sample design, but which can be addressed in the future. In addition, a relatively thin (200 nm) film was selected for study to minimize the number of domains illuminated during measurement. This led to a large initial volume fraction of *c* domains and minimal evolution of this quantity with electric field due to substrate clamping. Increasing the film thickness to reduce the clamping will provide further insights into domain wall behavior near grain boundaries, but will also increase the number of domains illuminated within each diffraction volume, further supporting the need for the combined real-space/reciprocal-space approach for mapping domain structure as advocated above.

Of particular interest in the future will be characterizing a much wider array of grain boundary angles and types to explore the fundamental materials science associated with reconfigurations of the domain states associated with microstructural features. There is currently a dearth of direct, *in situ* structural data at grain boundaries, contributing to an incomplete understanding of the nature of nonlinearity suppression mechanisms in ferroelectric films. It is still an open question as to whether variation of domain wall structure or mobility contributes to reduced 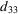 values observed at grain boundaries, but, here, differences in domain structure (variation of relative volume fractions of *a* and *c* domains) at the grain boundary were observed.
